# Helpful developments and technologies for school eye health programmes

**Published:** 2017-08-07

**Authors:** Priya Morjaria, Andrew Bastawrous

**Affiliations:** 1 Research Fellow and Public Health Optometrist: International Centre for Eye Health, London UK.; 2 Assistant Professor in International Eye Health: International Centre for Eye Health, London, UK. Co-Founder & CEO of The Peek Vision Foundation and its trading subsidiary, Peek Vision Ltd.

**School eye health programmes provide a unique opportunity to positively influence the health of 700 million children globally. The impact of school eye health (SEH) goes far beyond good vision—it encompasses education, social development and economic productivity.**

In all school eye health programmes, there are usually a number of factors which limit implementation, which can include a lack of trained personnel for screening, accurate diagnosis and acceptable treatment. The availabiliy of appropriate and affordable spectacle frames and lenses for children with refractive error, and access to specialist treatment for diagnosis and management of other eye conditions, are important resources that need to be accessible. New technology and innovative medical devices and software can be used at many stages in school eye health programmes. These innovations can make the programme more efficient and effective and can offer benefits to those running the programme as well as the children receiving care.

The following are essential to ensure that all children receive appropriate care:

- Visual acuity screening
- Simple eye examination
- Refractive error assessment
- Spectacle dispensing
- Identification of other eye conditions and referral
- Health education for children, parents and teachers.

Each task in a school eye health programme can affect the quality of care provided. Below, and in Table 1, we summarise some of the challenges and outline new developments that could assist in improving the quality and delivery of comprehensive school eye health. The list is not exhaustive and only gives a few examples of what is currently available.

## Screening

**Figure 1:**
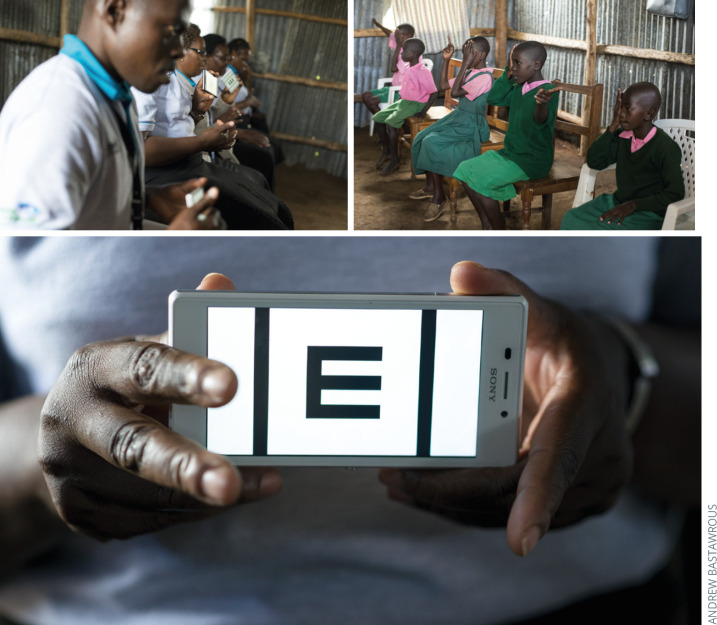
*Top:* Teachers screening children using Peek Acuity in a school in Trans Nzoia County, Kenya. *Above:* the Peek Visual Acuity app on a smartphone.

During screening, there are several challenges from the provider's perspective. The screening needs to be standardised in terms of the type of vision chart used, the training of the personnel who will screen, and the criteria for referral. We recommend using a single line optotype (see Figure 1, p. 30). Peek Acuity is a smartphone-based application which helps to standardise testing and referral and reduce the time taken to screen. It can be used to screen at a test distance of 2 m or 3 m, and the definition of pass or fail visual acuity screening (i.e. less than 6/9 or less than 6/12) is automated and can be adapted to the local programme (Figure 1). See more here: **https://www.youtube.com/watch?v=2l8RD-xsT30**

## Eye examination and refractive error assessment

**Figure 2:**
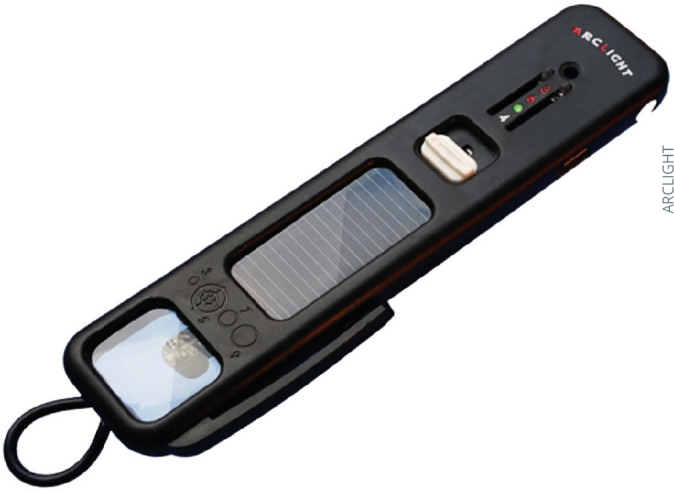
The ArcLight ophthalmoscope

These are crucial elements of a school eye health programme. Depending on the skills and qualifications of personnel available, these can be either two separate stages or be combined. To assist in an eye examination, low cost ophthalmoscopes are now available, including Optyse and ArcLight. These can also be used as torches to examine the anterior segment. ArcLight (Figure 2) has a solar panel to recharge the battery. Read more: **http://arclightscope.com/features/.** Initial assessment of the refractive error using an autorefractor (such as SmartVision or EyeNetra) can help to speed up refraction, but it is essential that this is followed by comprehensive refraction by a skilled practitioner.

## Spectacle dispensing

Dispensing and delivering spectacles pose further challenges, particularly when it comes to the availability of high-quality spectacle frames that are affordable, acceptable and appropriate for children (see article on page 33 for innovations in spectacles for children).

**Table 1:** Challenges in school eye health programmes and new developments

Challenges	New development
**Vision screening**	
Standardising the type of chart, distance of chart and definition of pass/fail acuity Minimising the subjectivity of the test Reducing the time taken Minimising false positives	Peek Acuity smartphone vision test
**Simple eye examination and refraction**	
Identification of false positive referrals Time taken to identify those that require refraction Skills required for refraction Equipment required for refraction Fundus examination Data entry and management	Refraction SmartVision – smartphone based autorefractor EyeNetra – Refraction and Electronic Medical Records SPOT – autorefractor SureSight Autorefractor Fundus examination ArcLight (low-cost ophthalmoscope) Optyse (low-cost ophthalmoscope) Smartphone ophthalmoscopes (Ophthalmic Docs Fundus) Management information systems (end-to-end systems) Peek School Screening - patient tracking and data entry Orbis REACH
**Dispensing and health education**	
Over-prescribing Lack of frame choices No health education/information given to child or parent Time taken to receive spectacles Cost of spectacles Quality of spectacles: lenses and frames Data entry and management	Ready-made spectacles Ready-to-clip spectacles PeekSim images Voice messages SMS to carers
**Other eye conditions**	
Skills required to identify eye conditions Equipment required	Arclight (anterior segment loupe also) Smartphone ophthalmoscopes (multiple)
**Specialist referral and health education**	
Referral pathway Clear referral criteria Access to service Awareness of importance of referral by parents/carers	Voice messages SMS Peek simulation images Peek School Screening System

## Treatment of other eye conditions and specialist referral

The following children need to have a detailed eye examination, including examination of the posterior segment: those with strabismus, corneal opacities, or high degrees of refractive error, and those for whom visual acuity does not improve to normal with refraction. Low-cost ophthalmoscopes, such as ArcLight or Optyse, can also be used for this purpose. After identification, these children need to be referred for further examination as appropriate, e.g., for routine/urgent ophthalmologist review or cycloplegic refraction.

## Health education

The final stage in the process is health education of parents/carers, as it is vital that they know about the results of the screening and eye examination of their child, and the action required. This is usually done by giving the child an information sheet or pamphlet to take home for their parents, but this can be challenging where many adults are not literate or where multiple languages are used. Software, such as the Peek School Screening System, can address this by sending SMS or pre-recorded voice messages to parents/carers. Peek can also generate photographs which show how the world appears to a child with uncorrected refractive error (simulation images), which can also be sent to parents.

## Management information system (MIS) software

Management information system (MIS) software, such as Orbis' REACHSoft, capture data for planning, implementation, and monitoring of field activities. The data generated can then be analysed and used to better understand local service delivery challenges.

**Figure 3:**
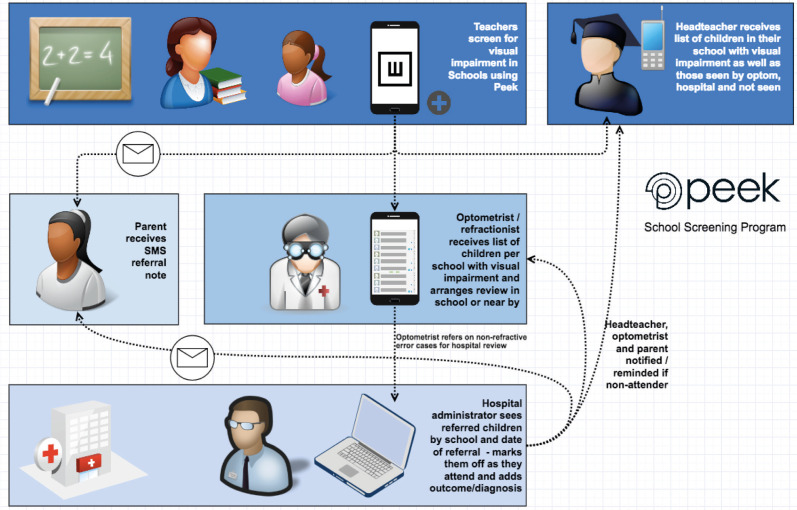
The Peek School Screening System and how it can be used to track children through the system and for health education of parents/carers

Another example of a MIS system is the Peek School Screening System (Figure 3), which works in tandem with the Peek Acuity App. It is integrated with software that tracks children as they move through each stage in the comprehensive school eye health programme (Figure 3). It can, for example, automatically create a referral for children who have failed screening or have been identified as needing more specialist care, and communicate this to staff at the base hospital or vision centre. The system is also able to capture parents' mobile numbers so they can be kept informed and also receive reminders of follow-up appointments, etc. The software allows children to be tracked at each stage in the process, and generates data that can be used to monitor the programme in real-time, identifying bottlenecks early in the process.

Table 2 summarises how management information system software can be of benefit to programme managers as well as children and their families.

**Table 2:** The benefits of management information software to programme managers, children and parents

Who benefits	Benefits
Programme managers	Visual acuity screening is quicker and easier with standardised optotypes or Peek Visual Acuity App Potential to reduce the burden on specialist eye services Better accountability as bottlenecks in the system can be detected early and resolved in a timely manner Provides a system that enables continuous improvement Alignment of different partners around standardised outcomes Easier reporting systems and greater accountability
Children and their parents	Reduced waiting for screening, eye examination/refraction and spectacle dispensing Empathy from teachers, parents and peers with increased awareness and knowledge about ocular conditions Better communication with decision makers with an opportunity to have concerns addressed Less travel for further review/treatment Less stigma about the use of spectacles or eye treatments Better vision

